# Molecular Tumour Board (MTB): From Standard Therapy to Precision Medicine

**DOI:** 10.3390/jcm12206666

**Published:** 2023-10-21

**Authors:** Zelmira Ballatore, Francesco Bozzi, Sara Cardea, Francesco Domenico Savino, Antonella Migliore, Valentina Tarantino, Natalia Chiodi, Elisa Ambrosini, Francesca Bianchi, Gaia Goteri, Alessandra Filosa, Francesca Barbisan, Elisa Bartoli, Roberto Papa, Rossana Berardi

**Affiliations:** 1 Department of Medical Oncology, AOU delle Marche, 60126 Ancona, Italy; zelmira.ballatore@gmail.com (Z.B.); valentina.tarantino@ospedaliriuniti.marche.it (V.T.); 2 Medical Oncology, Università Politecnica delle Marche, 60126 Ancona, Italy; francescobozzi1996@gmail.com (F.B.); s1103488@pm.univpm.it (S.C.); francescodsavino@gmail.com (F.D.S.); s1102265@pm.univpm.it (A.M.); n.chiodi@pm.univpm.it (N.C.); e.ambrosini@pm.univpm.it (E.A.); f.bianchi@staff.univpm.it (F.B.); 3 Anatomia Patologica, AOU delle Marche, Università Politecnica delle Marche, 60126 Ancona, Italy; g.goteri@staff.univpm.it (G.G.); alessandrafilosa@yahoo.it (A.F.); francesca.barbisan@ospedaliriuniti.marche.it (F.B.); elisa.bartoli@ospedaliriuniti.marche.it (E.B.); 4 Quality, Risk Management and Health Technology Innovation Unit, Department of Staff, AOU delle Marche, 60126 Ancona, Italy; roberto.papa@ospedaliriuniti.marche.it

**Keywords:** molecular tumor board, precision medicine, NGS

## Abstract

**Background**: In the metastatic setting, cancer patients may not benefit from standard care regimes and their diseases undergo drug resistance due to tumour cell heterogeneity and genomic landscape complexity. In recent years, there have been several attempts to personalise the diagnostic-therapeutic path and to propose novel strategies based on not only histological test results but also on each patient’s clinical history and molecular biology. Profiling molecular tests allows physicians to investigate the single tumour genomic landscape and to promote targeted approaches. The Molecular Tumour Board (MTB) is a multidisciplinary committee dedicated to selecting individualised and targeted therapeutic strategies appropriate for patients suffering from diseases that present resistance to standard care. **Materials and Methods**: Our MTB settled in “Azienda Ospedaliero Universitaria delle Marche”, Ancona (AN), Italy, and includes oncologists, molecular biologists, geneticists, and other specialists. Clinical cases are referred by physicians to the MTB, through the Cancer and Research Centre of the Marche Region (CORM), through a telemedicine platform. Four possible molecular profiles are available: FoundationOne^®^ CDx e FoundationOne^®^Liquid CDx and two local Next Generation Sequencing (NGS) panels, with 16 DNA genes and 10 RNA genes respectively. The resulting genetic mutations and their analyses are evaluated by all the members of the Board and a report for each patient is provided with medical recommendations. **Results**: from June 2021 to May 2023, we collected data from 97 referral patients (M: 49, F: 48). The mean age was 60.6 years (range 22–83 years). 90 cases were approved for testing. Only seven patients were not eligible for genomic profiling. In two patients who were eligible, molecular profiling was not performed because a tissue sample was not available. Off-label therapy was recommended for three patients. 5% of cases (5/88) showed addressable driver mutations associated with an existing targeted therapy and were immediately enrolled. **Conclusions**: MTB presents a powerful tool for offering precise medical goals. Our Department of Clinical Oncology also takes advantage of the important role of multidisciplinary teams, through the establishment of CORM and MTB meetings, within which there is the chance to perform NGS-based analyses. It will be important in the future to implement the use of genomic profiling to improve personalised care and to guide the choice of suitable therapies and more appropriate management of patients.

## 1. Introduction

Clinical oncology is changing rapidly and dramatically thanks to the development of innovative technologies and the advances in genetics and genomics, which have offered new opportunities for personalised treatment to cancer patients revolutionising their therapeutic strategy and outcomes [1].

In addition to the morphologic and histologic factors of a tumour, its genomic profile may lead to more precise patient selection, in which specific molecular alterations in the tumour become the target of individualised treatment, thus consolidating the well-known concept of treatment personalisation also using the new model of mutational oncology [2].

Precision medicine represents a new era of medicine and considers the variability of the population based on genetic, epigenetic, socio-environmental, and lifestyle factors to define the best therapeutic strategy [3,4].

The goal of precision oncology is achieved through multiplex molecular testing, including Next Generation Sequencing (NGS) [5]. This high-throughput technology is being increasingly integrated into routine clinical cancer care, providing patients and their oncologists with molecular characterisation of their tumours and a promise of personalised therapy. 

NGS technology is becoming cheaper, faster, and more available by the day6. Oncologists should be familiar with technical aspects of NGS to promote the most appropriate and cost-effective testing platform. However, the need to manage large volumes of data [6,7], as well as the interpretation of the molecular findings and access to drugs/clinical trials is still challenging [1]. 

Moreover, fair access, with homogeneous criteria, and economic sustainability of the genomic tests which may be indicated, should be warranted within clinical practice. Furthermore, translating and interpreting the complex genetic and molecular features of the tumour into information that medical oncologists can use to propose the most appropriate and personalised treatment is crucial. 

All these elements represent the rationale behind the implementation of the Molecular Tumour Board (MTB) [5]. 

MTB includes specialists with expertise in different medical fields, from translational research to computational biology, with the aim of integrating a comprehensive review of the patient’s characteristics, including clinical history, imaging, pathology, laboratory results, and molecular profiling [3]. Attendees include medical oncologists, pharmacists, bioinformatics, researchers, biologists, geneticists, and pathologists [8]. MTB can be effective in processing comprehensive genomic profiling (CGP) results and can permit the identification of customised, often multi-agent, treatment-to-target driver alterations, taking into context biomarkers of resistance, drug interactions, and clinical history [5].

Therefore, the goal of the MTB is to develop an N-of-One treatment plan that could be initiated by the patient’s physician under the auspices of a master protocol, with the assistance of clinical trial coordinators/navigators and medication acquisition specialists to facilitate drug availability [3].

In this manuscript, we describe a single institutional experience of the Molecular Tumour Board at the AOU (Azienda Ospedaliero Universitaria) of the Marche Region, focusing on the innovative procedure adopted and also investigating the profiled patients that, in this context, actually benefit from precision medicine [3,4]. A further aim is to provide a contribution to the efficient handling of the practical issues deriving from 2 years of MTB meetings.

## 2. Materials and Methods

### 2.1. Blueprint Service

Our multidisciplinary team at the AOU of the Marche Region includes medical oncologists, pathologists, molecular biologists, pharmacists, geneticists, researchers, and a data manager.

The “Blueprint Service” model was used to design the MTB service introducing innovative elements able to improve the relationship with patients and consequently the clinical outcomes. Blueprint Service is an operational planning tool that provides indications on the way the service will be provided, indicating the necessary actions and support systems required to provide the service. The model shows the channel through which the service works, and it allows users to periodically return to the project to improve it over time as the organisation and its operational contents change.

**Figure 1:**
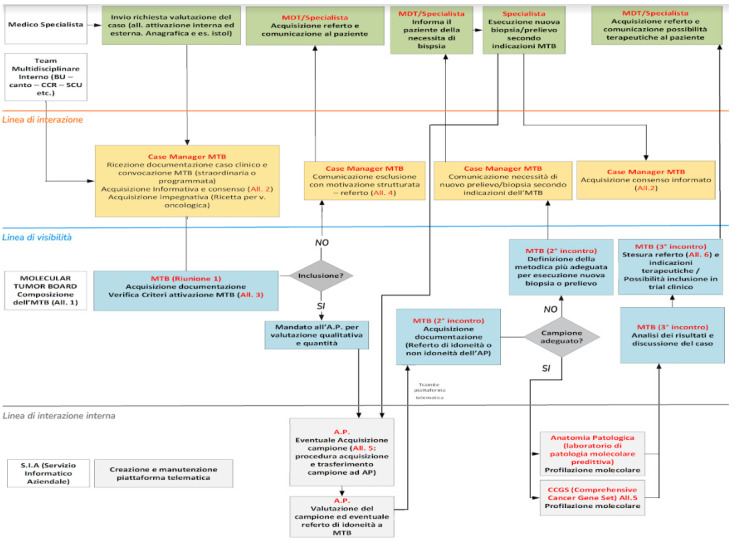
Blueprint service. Operational planning tool that provides guidance on how a service will be provided.

Processing steps to setting up the “Blueprint Service” include the identification of the process to be mapped, identification of medical specialists or a multidisciplinary team who take care of the patient as a stakeholder, and process mapping by a stakeholder (Figure 1).

### 2.2. CORM and MTB

Cancer and Research Centre of Marche (CORM—Centro Oncologico e di Ricerca delle Marche) is a comprehensive cancer center established at the Department of Oncology of the AOU of the Marche Region in 2021 under the patronage of the Ministry of Health in April 2021. It includes an informatics platform of telemedicine with a dedicated website (www.corm-marche.it, accessed on 3 August 2023) that permits the connection between our hospital and 13 different Oncology Hospitals on the periphery of the Marche region [9]. In this way, the displacement of patients has been limited to those eligible for an experimental protocol.

Once the physician has completed the submission on the platform, our MTB multidisciplinary team gathers and evaluates the eligibility of each case. This happens through periodic meetings held for 1 h every 2 weeks. We started the discussions of the clinical cases on 9 June 2021 and analysed the activity performed until the end of 2022. These meetings are accredited for continuing medical education for all health professionals, including medical oncologists, biologists, pathologists, pharmacists, and geneticists. All physicians who activate the evaluation through MTB are allowed to participate in the meetings, which are held both in person and remotely.

In these sessions, a variety of patients’ needs are discussed, and once a specific case study has been completely illustrated, decisions regarding management and the best diagnostic and therapeutic path are taken. During the multidisciplinary discussions, we establish which molecular test can be proposed for which patient, according to the European (ESMO—European Society for Medical Oncology) and National (AIOM—Italian Association of Medical Oncology) guidelines. Italy complied with the international guidelines with the law No. 233 of 29 December 2021 “Istituzione dei Molecular Tumor Board e individuazione dei centri specialistici per l’esecuzione dei test per la profilazione genomica Next Generation Sequencing (NGS)”, therefore since 2022 we have acted according to the national law, too. ESMO recommends using tumour multigene NGS in patients presenting with advanced non-squamous NSCLC, prostate, ovarian cancers, cholangiocarcinoma and primary of unknown origin. Large panels of genes can be used if they generate only a modest increase in the overall cost, drugs included. In colorectal cancers, NGS can be an alternative to PCR-based tests, if it is not associated with extra cost [10].

At the end of the meetings, a written report containing all the patient’s characteristics (e.g., age, gender, physician’s name, and diagnosis) is formulated and a copy is sent to all of the core team by the IT platform. The report summarises the therapeutic options proposed by the MTB, approved drugs, off-label treatments, or clinical trials depending on molecular findings.

Patients have to preliminarily sign a dedicated informed consent regarding the diagnostic and therapeutic recommendations [6].

The aims of CORM are as follows:

- Support and consolidation of the regional oncology network;
- Encouragement and development of clinical and translational research, including phase I trials with innovative drugs that need an Italian Regulatory Agency of Drugs (AIFA) certification. The Department of Oncology of AOU of Marche is the only active Phase I Centre in the Marche region;
- Support of research in oncological genetics for hereditary cancers. The AOU of the Marche region also includes the Highly Specialised Regional Reference Centre in cancer genetics and this represents a benefit for all the regional hospitals;
- Promotion and consolidation of cancer diagnostic and therapeutic pathways;
- Promotion of the regional model of PDTA.

### 2.3. Molecular Testing

MTB chooses between “singleplex” tests, able to analyse specific molecular targets (e.g., through RealTime-PCR methods), or “multiplex” technologies if there are different biomarkers for different patients to be evaluated (e.g., NGS). In this last case, the analyses are performed on the nucleic acids (DNA or RNA) extractable from histological tumour samples (surgical resections or tumour biopsies) or from circulating tumour cells (liquid biopsies) using a test based on the NGS method.

The MTB of the Marche Region has access to the following NGS gene panels and analyses:

(1) Myriapod® NGS Cancer panel DNA Illumina® (CE IVD, Diatech Pharmacogenetics SRL, Jesi (AN), Italy), a DNA Gene Panel with 16 genes. The test allows for the identification of single nucleotide variants (SNV) and insertions and deletions (indels) in 16 genes of clinical-diagnostic relevance in major cancers (ALK, BRAF, EGFR, ERBB2, FGFR3, HRAS, IDH1, IDH2, KIT, KRAS, MET, NRAS, PDGFRA, PIK3CA, RET, ROS1), starting from DNA extracted from FFPE (formalin-fixed paraffin-embedded) tissue and ctDNA;
(2) Myriapod® NGS Cancer Panel RNA Illumina® (CE IVD), a RNA Gene Panel with 10 genes. This is the panel dedicated to the study of gene fusions on 10 targets of interest (ALK, ROS1, RET, MET, PPARG, FGFR2, FGFR3, NTRK1, NTRK2, NTRK3) for the prediction of response to oncological drugs, starting from RNA extracted from FFPE tissue;
(3) Real-Time RT-PCR NTRK analysis, using EasyPGX® ready NTRK Fusion (Diatech Pharmacogenetics SRL, Jesi (AN), Italy), and immune-histochemistry analysis, such as IHC DMMR and IHC PDL1;
(4) FoundationOne® CDx, a DNA single tissue-based test with 324 genes. This is the first FDA-approved tissue-based broad companion diagnostic (CDx) that is clinically and analytically validated for all solid tumours. The test is designed to provide physicians with clinically actionable information to consider appropriate therapies for patients and to understand results with evidence of resistance based on the individual genomic profile of each patient’s cancer. Test results include microsatellite instability (MSI) and tumour mutational burden (TMB) to help inform immunotherapy decisions and loss of heterozygosity (LOH) for ovarian cancer patients;
(5) FoundationOne®Liquid CDx analyses 324 genes from circulating cell-free DNA and is FDA-approved to report short variants in 311 genes.

Nucleic acid extraction of each sample is performed manually using QIAamp^®^ DNA Mini kit (Qiagen, Hilden, Germany) or automatically with the automatic extractor Magcore and MagCore^®^Genomic DNA FFPE One-tep kit (Diatech Pharmacogenetics, Jesi, Italy).

The quantification of nucleid acid extracted is performed using EasyPgx^®^ (Diatech Pharmacogenetics) and the analysis of qPCR reaction data for determination of DNA concentration of FFPE samples and its quality is performed with EasyPgx^®^ Analysis Software version 4.0.14 (Diatech Pharmacogenetics). Qubit^TM^ 1× dsDNA HS Assay Kit (Thermo Fisher Scientific, Waltham, MA, USA) is the kit used for the final quantification of the pool to be sequenced.

The bioinformatics platform used for the analysis of sequencing is Myriapod^®^ NGS Data Analysis Software version 5.0.8 NG900-SW 5.0.8 and Myriapod^®^ NGS Workstation NG900-HD (Diatech Pharmacogenetics).

All NGS tests were performed on the MiSeq^®^ System Illumina instrument. Qscore, Estimated Yield, Cluster Density, and Clusters Passing filter are the parameters used to evaluate the quality of sequencing while Uniformity, % reads on target, and % regions below threshold are the parameters used to evaluate the quality of sequencing of each sample and its related variants calls. Somatic variants with allele frequency (VAF) > 5% and a minimum coverage of 500× are considered reliable.

## 3. Results

Between 8 June 2021 and 31 May 2023, a total of 97 patients were pre-screened to possibly carry out molecular profiling. Of them, 7 (7/97, 7.2%) received no indications to undergo molecular profiling, whilst 24 (24/86, 27.9%) obtained a positive test for ≥1 gene alteration.

### 3.1. Patients’ Characteristics

97 tissue or blood samples of cancer patients were collected and discussed with a mean age of 60.6 years [range: 22, 83]. Of these, 49 were males with a mean age of 62.9 years [range: 29–83 years] and 48 were females with a mean age of 58.4 years [range: 22–82 years]. 32 out of the 97 patients, were presented by internal oncologists, while the remaining 65 cases were presented by oncologists from various hospitals in the Marche region. All patients had received a median of two prior therapies. For 11 patients, given the scarcity of documentation received, it was not possible to trace the therapeutic lines previously performed (Table 1).

**Figure 2:**
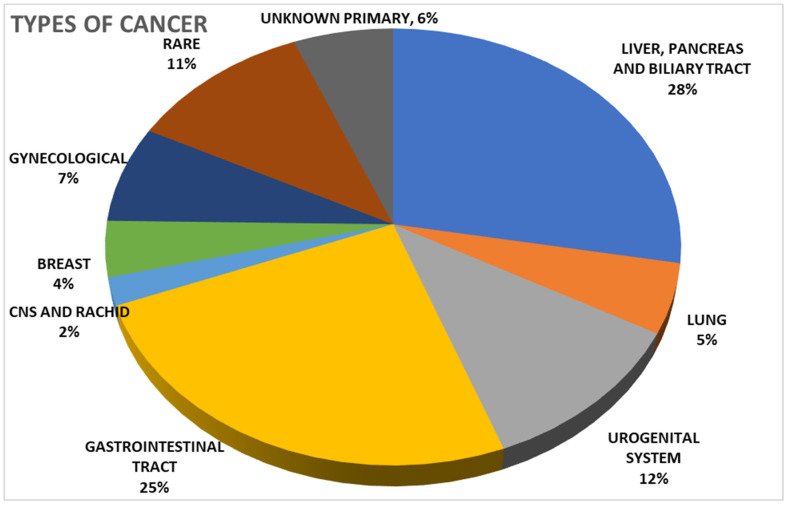
Types of Cancer.

In total, 27 out of the 97 discussed cases were patients with pancreatic and biliary tract cancers, of which 5 had lung tumours, 11 had tumours of the urogenital system, and 24 had gastrointestinal tumours, while 2 were affected by CNS and rachis tumours, 4 by breast cancers, 7 by gynaecological cancers, 11 by rare tumours (sarcomas, GIST, thymus cancers), and 6 by a primary cancer of unknown origin (Figure 2).

### 3.2. Genomic Profiling

Seventeen patients (17/88, 19.3%) received FoundationOne^®^CDx and FoundationOneLiquid^®^CDx, 3 patients (3/88, 3.4%) were tested with the panel FoundationOne^®^Heme as appropriate, 5 (5/88, 5.7%) patients were screened only with FoundationOne^®^Heme. In particular, 6 out of the 22 patients who received the FoundationOne Test, were patients with cholangiocarcinoma, 3 with colorectal cancer, 3 with rare tumors, 2 with lung cancer, 2 with breast cancer, 2 with ovarian cancer, 2 with a primary cancer of unknown origin and 2 with pancreatic cancer. Finally, 3 patients with sarcoma were tested with Foundation Heme panel.

**Figure 3:**
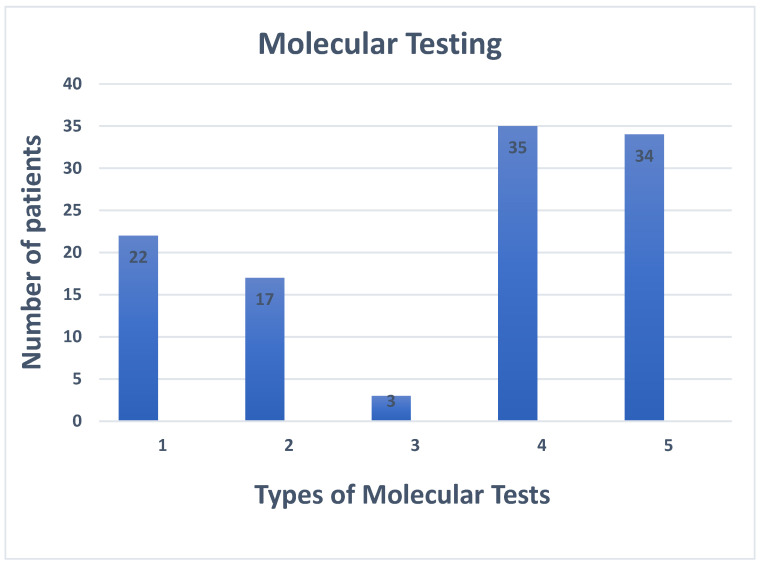
Types of molecular tests. Legend: 1. Patients tested with FoundationOne^®^CDx 2. Patients tested with FoundationOneLiquid^®^CDx 3. Patients tested with FoundationOne^®^Heme. 4. Patients tested with local panel (10 genes tested). 5. Patients tested with local panel (16 genes tested):.

Furthermore, 13 (13/88, 14.8%) patients were tested with our local panel of 10 genes (RNA), and 12 (12/88, 13.6%) patients were tested with another of our local panel of 16 genes (DNA). Twenty-two patients were tested with both local panels and, finally, 16 patients were tested for alterations such as HRD (Homologous recombination deficiency) and somatic and germline BRCA alterations. Five out of these 16 patients (5/16, 31.3%), had prostate cancer and were tested for somatic alterations, three patients (3/21, 18.8%) with pancreatic cancer were tested for somatic alterations. The remaining patients were divided between ovarian (5/16, 31.3%) and breast cancer (3/16, 18.8%), and were tested for somatic and germline alterations, respectively. A pathogenic BRCA mutation was found in only one patient. (Figure 3).

**Figure 4:**
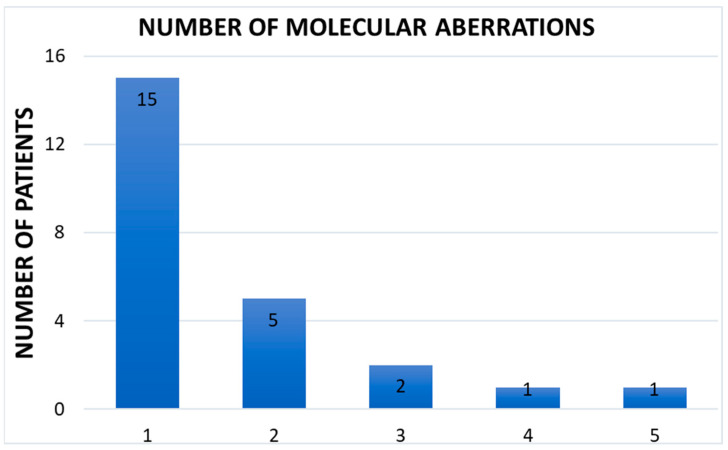
Number of molecular aberrations.

A total of 36 gene mutations (IDH1, TP53, EGFR, FGFR2, PIK3CA, PIK3R1, PTEN, Myc, KRAS, MET, MDM2, and other) were found in 24 patients and 9 of them presented ≥2 gene alterations (Figure 4). 15 patients did not have enough tissue samples to perform the analysis, and the others did not present molecular alterations. According to the molecular profiling, 8 patients showed actionable mutations: five patients benefited from target therapy. Of these, 3 patients have been enrolled in clinical trials, while 2 patients have undergone treatments approved by AIFA. For the remnant three patients, our MTB recommended compassionate treatment for histology not yet approved by AIFA (Ivosidenib for a patient with cholangiocarcinoma presented IDH1 mutation, Selpercatinib for a patient with adenocarcinoma of the esophagus presented RET mutation and then Olaparib/Niraparib for HRD).

Seven patients were not eligible for molecular profiling owing to their poor prognosis. In two cases where molecular profiling was indicated, it was not performed owing to the lack of a tissue sample.

## 4. Conclusions

Molecular profiling tests, their complex data, and treatment opportunities for cancer patients are continually increasing. The medical oncology system operates in an environment populated with immense amounts of new information about molecular biology, biomarkers, and new treatments. Clinicians and molecular biologists themselves are separately less efficient in gathering and elaborating the vast amount of information [1,6,7]. Our study showed the development of a new model of MTB. This is an innovative approach that offers the most recent molecular profiling technologies to a variety of eligible patients throughout the whole region, thanks to and through the virtual IT platform, CORM. Advances in genomic technologies, including NGS, have improved the management of cancer patients. The identification of mutational or enriched oncogenes targets in the diseases of individual patients may lead to specific biological therapies, resulting in a more precision medicine-oriented approach. We compared some of the outcome tendencies between patients treated with MTB-directed therapy alongside those treated with standard care regimes: the majority of patients experienced improved progression-free survival (PFS) compared with the previous treatment. MTB-directed targeted therapy may represent a future strategy to improve the survival rate of patients with advanced cancer [11].

Our MTB service worked as a “gate-keeper”, avoiding unnecessary assessing procedures and their relative costs.

As a part of the MTB protocol, patients were enrolled in targeted therapy in a clinical trial setting, after identifying a biomarker-based study active at our Department or in other sites, or they were candidates for off-label treatments if a drug against the driver mutations was available for a different indication.

Patient eligibility and the chance of off-label or compassionate usage of the specific drug depended on whether the patients received the targeted therapy or were enrolled in the trial.

Our MTB methodology could help to translate increasingly complex genetic information into patient-centered clinical decisions, thereby leading precision oncology into daily practice. This experience evaluates current knowledge and needs and provides recommendations that may serve as a roadmap for successful MTB implementation specifically referring to the cooperation strategies between several different professional figures, as well as useful data regarding possible unconventional disease-drug pairings in advanced patients’ cancer settings.

Limitations of this study include the limited sample of enrolled patients and diversity in patient races. Furthermore, hospital centers that refer to our MTB, because of the initial inexperience, brought to our attention heavily pre-treated patients with a poor prognosis, not qualifying for eligibility. Therefore, they could not benefit from the inclusion into clinical trials and from treatment with targeted therapies. Hence a low actionability of our study, which however appears to be in line with that of other Italian and European centers. In addition, our patients benefited from a limited range of targets due to the narrow amount of assessed genes by our local panels, although we are currently implementing a wider study, thus further results are awaited in the near future. Large genetic panels were not administrable in all the patients since no drugs were potentially available in their settings against a considerable expected cost. That is exactly why it is essential to broaden the amount of information currently available on novel specific synergies that could evolve into therapeutic strategies.

In conclusion, MTB has given us an opportunity for continuous learning about the application of precision medicine and improved patient outcomes. A well-designed MTB system will evolve along with the technology to ensure that patients receive the best possible treatment without unnecessary costs or risks. It could also improve clinical knowledge and skills, to help guide physicians in their decisions. In conclusion, we hope that our experience could be useful to implement the management of advanced complex cancer patients in daily clinical routine and may lead to a more patient-oriented precision medicine approach in the near future.

## Figures and Tables

**Table 1:** Patients’ characteristics.

Period	June 2021–May 2023
Number of meetings	~50
Age	60.6 Years Range, 22–83 years
Gender, N (%)	Male, 49 (50.5%) Female, 48 (49.5%)
Number of Physicians who presented ≥ 1 case	35
Diagnosis, N	
Breast cancers	4
Pancreas and biliary tract cancers	27
Gastrointestinal cancers	24
Lung cancers	5
Gynaecological cancers	7
Rare cancers (sarcoma, NET)	11
Urogenital System cancers	11
CNS and Rachid cancers	2
Cancer of Unknown Primary (CUP)	6

## Data Availability

Data is unavailable due to privacy. Data will be share on specific request.
